# The research trend on neurobrucellosis over the past 30 years (1993–2023): a bibliometric and visualization analysis

**DOI:** 10.3389/fneur.2024.1349530

**Published:** 2024-09-23

**Authors:** Lanting Yang, Wei Pan, Qiong Cai, Mingyang An, Chunjie Wang, Xilong Pan

**Affiliations:** ^1^Department of Social Medicine and Health Education, School of Public Health, Peking University, Beijing, China; ^2^Faculty of Arts, The University of Melbourne, Melbourne, VIC, Australia

**Keywords:** neurobrucellosis, CiteSpace, VOSviewer, cerebrospinal fluid, antibiotic therapy

## Abstract

**Background:**

Brucellosis is a zoonotic disease caused by Brucella infection, which is common in pastoral areas. Neurological involvement in brucellosis is relatively rare. But since 1993, continuous studies have been reporting neurological complications of brucellosis, collectively referred to as neurobrucellosis. A bibliometric analysis of existing literature outlines current research progress and gaps and provides guidance for the clinical treatment of neurobrucellosis, promoting patient health in the process of guiding clinical practice, and improving their quality of life.

**Methods:**

CiteSpace and VOSviewer are software tools to visualize research trends and networks. By selecting specific areas of interest and configuring the right parameters, the tools can visualize past research data. The study retrieved the literature from the Web of Science Core Collection Database and downloaded it in plain text file format. Citespace6.1.6, VOSviewer v1.6.20, and Microsoft Excel 16.59 were used for analyzing the following terms: countries, institutions, authors’ cooperation, journals, keywords, and co-citation.

**Results:**

There are eight key results. (1) The publication volume shows a general upward trend. (2) Turkey is the country with the highest publication volume and contributing institutions. (3) Giambartolomei GH, Gul HC, and Namiduru M are the authors with the highest number of publications. (4) Neurology is the journal that published the highest number of related articles (*n* = 12). (5) “Diagnosis,” “meningitis,” and “features” are the top three frequently occurring keywords. (6) Keyword clusters show “antibiotic therapy” and “cerebrospinal fluid” have future study value. (7) The burst analysis of the keywords also indicates that “cerebrospinal fluid” may become a prominent keyword in future research. (8) The co-citation analysis concludes three categories of the cited articles, which are diagnosis, therapy, and complications, indicating the past research direction.

**Conclusion:**

This study highlights the complexity of neurobrucellosis, presenting the need for advanced diagnostic techniques and multifaceted treatment approaches. While antibiotics remain the cornerstone of therapy, the use of corticosteroids to mitigate inflammatory responses shows promise, albeit with concerns about potential sequelae and relapse. Future research should focus on refining therapeutic strategies that address both the direct effects of infection and the broader immunological impacts to improve patient outcomes and quality of life.

## 1 Introduction

Brucellosis is one of the most common zoonotic diseases worldwide. It is typically transmitted through direct contact with infected animals or consumption of unpasteurized products. Since 1993, there have been continuous studies reporting neurological complications of brucellosis, collectively referred to as neurobrucellosis, but only approximately 5% of brucellosis patients experience nervous system involvement (1). The pathogenesis of neurobrucellosis involves the invasion of Brucella bacteria into the blood, reaching the meninges and nervous system. The damage to both the peripheral and central nervous systems is predominantly attributed to indirect immune mechanisms (2–4). Frequent complications involving the central nervous system include acute meningitis, myelitis, strokes, subarachnoid hemorrhages, seizures, and cranial nerve involvement, especially the vestibulocochlear nerve (3, 5, 6). Cognitive disorders, psychiatric symptoms, and pituitary region involvement are rare but documented manifestations (6, 7). Complications involving the peripheral nervous system include Guillain Barre syndrome, sciatica, and polyradiculopathy (8–11). Clinical manifestations of neurobrucellosis vary widely, including headaches, vision and hearing impairments, confusion, incontinence, behavioral changes, muscle weakness, and neck rigidity (3, 12, 13). Diagnosis relies on a combination of suggestive symptoms, positive cerebrospinal fluid (CSF) culture or Brucella IgG agglutination in blood, lymphocytic pleocytosis, increased CSF protein, and response to specific antibiotics (2). Although there are many detection methods, the diagnosis is challenging due to its variable manifestations and delayed clinical recognition. Neurobrucellosis is usually diagnosed within 2–12 months after the symptom onset (6, 12).

Most existing articles on neurobrucellosis are primarily case reports, and the sample sizes are generally small, with the majority not exceeding 10 cases (14–16). These articles typically study manifestations, diagnosis, and treatment therapy of neurobrucellosis. There are also a few articles that focus on the immunopathology of neurobrucellosis, which provide valuable insights into the disease’s pathophysiology, diagnostic biomarkers, and therapeutic strategies (17–19). However, there are virtually no comprehensive reviews that integrate and analyze these case studies statistically to track advancements in specific areas such as pathophysiology, diagnostic biomarkers, and treatment therapies. This lack of detailed synthesis contributes to a limited understanding of the existing research gaps in neurobrucellosis.

Web of Science Core Collection Database provides a comprehensive view of scholarly literature. Using this database can ensure the comprehensiveness of literature searches. CiteSpace is a software used for the visualization and analysis of trends and patterns in scientific literature. This study will use CiteSpace and VOSviewer to conduct a bibliometric analysis of the articles related to neurobrucellosis within the Web of Science Core Collection. The aim is to systematically review the existing literature on neurobrucellosis, providing a comprehensive overview that identifies current research trends and gaps. This will guide future studies and provide guidance for the diagnosis and clinical treatment of neurobrucellosis, ultimately aiming to improve the quality of life of patients.

## 2 Methods

### 2.1 Data source and search strategy

The researcher retrieved literature on the database, the Web of Science Core collaboration. The searching method is topic searching from January 1, 1993, to October 31, 2023, including search titles, abstracts, author, and keywords. The keywords are Brucellosis, including “Mediterranean fever,” “Undulant fever,” “Malta fever,” and Neurological Disease, including “nervous system disorders,” “Nervous system disease” and “neurological disorders.” To ensure the comprehensiveness of the search, this study also searched for related keywords including “nervous system brucellosis” and “neurobrucellosis.” The search and data were reported on October 31, 2023.

### 2.2 Inclusion and exclusion criteria

The inclusion and exclusion of studies were based on the filters of the WOS database. The studies that met the following criteria were included: (1) articles published in the period from January 1, 1993, to October 31, 2023; (2) articles about Neurobrucellosis; (3) original Articles, Review articles and book chapters; (4) published in English. Exclusion criteria were: (1) duplicate publications; (2) early access; (3) letters, abstracts, conference presentations, and unpublished manuscripts; (4) articles not related to the topic of neurobrucellosis.

Relevant articles were selected for the final analysis by reviewing the abstracts and titles.

### 2.3 Data export and standardization

The full record and cited references of selected articles were exported in plain text format and imported to Citespace 6.1.6 and VOSviewer v1.6.20. Keywords with the same meaning were standardized: “nervous system brucellosis” is replaced by “Neurobrucellosis”; “Nervous system diseases” or “Nervous system disorders” are replaced by “neurological disease”; “CNS” is replaced by “Central Nervous System.”

### 2.4 Bibliometric software

Bibliometric analysis is a research method used to quantitatively analyze scientific literature (20). CiteSpace and VOSviewer are software tools designed for the analysis and visualization of scientific literature and citation data (21). CiteSpace is primarily used to detect critical points and visualize emerging trends of scientific studies in a specific area (22). It is effective for identifying keywords, key authors, and institutions, often represented through cluster analyses and timeline views. VOSviewer specializes in creating network maps of scientific literature, enabling users to visually explore bibliometric networks, including journals, researchers, or individual articles, which helps to illustrate the relationships based on co-authors, co-citations, or bibliographic coupling (23). Users typically import data from databases and then utilize the software’s analytical and visual capabilities to interpret the data and generate insights about how scholarly activities evolve. This study will use Citespace 6.1.6 and VOSviewer v1.6.20 to visualize the development of scientific literature on Neurobrucellosis.

### 2.5 Data analysis

To analyze the development of neurobrucellosis research, data were systematically collected from selected articles fitting specific inclusion/exclusion criteria. The country and keyword clusters were analyzed in CiteSpace. Co-authorship, keywords co-occurrence, and co-citation analyses were conducted using VOSviewer. The specification settings of CiteSpace are as follows: (1) the Time slicing is from January 1993 to December 2023, with each slice representing 1 year; (2) Text Source includes Title, Abstract, Author Keywords (DE), and Keywords Plus (ID); (3) Node Types include countries for analysis of authors’ countries of origin, and keywords for keyword clusters and burst analysis; (4) Link Strength is set to Cosine, and Link Scope to Within Slices; (5) Selection Criteria employs the g-index in each slice, and the scale factor K is set at 25, which is optimal to present a moderate amount of data clearly, facilitating the identification of significant trends, clusters, and connections within the data. The specification settings for VOSviewer, which differ due to the nature of analyses performed, will be detailed in the sections on co-authorship, keywords co-occurrence, and co-citation, underscoring the distinct configurations necessary for these analyses.

## 3 Results

### 3.1 Search results

**Figure 1:**
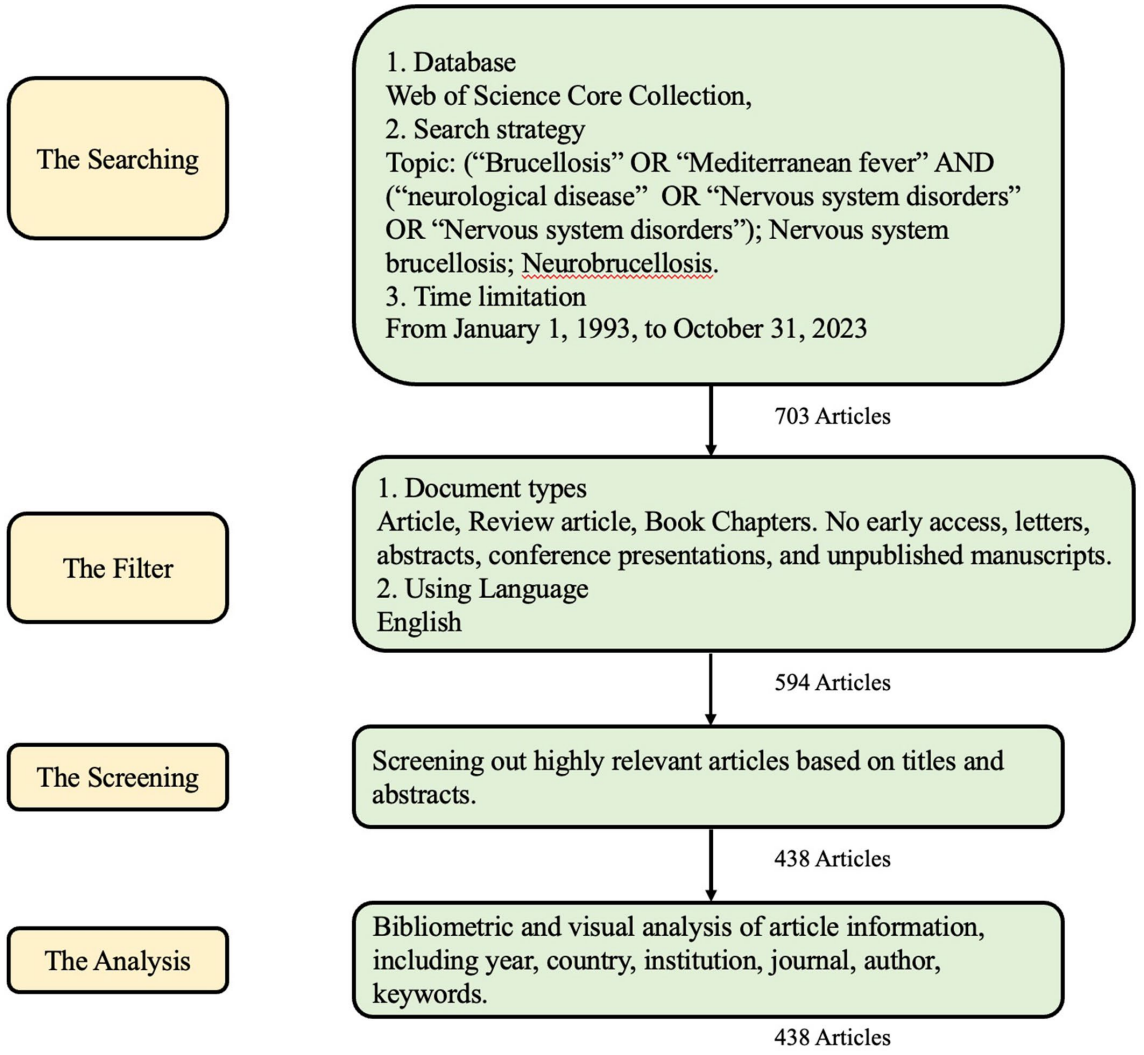
Searching flow diagram.

**Figure 2:**
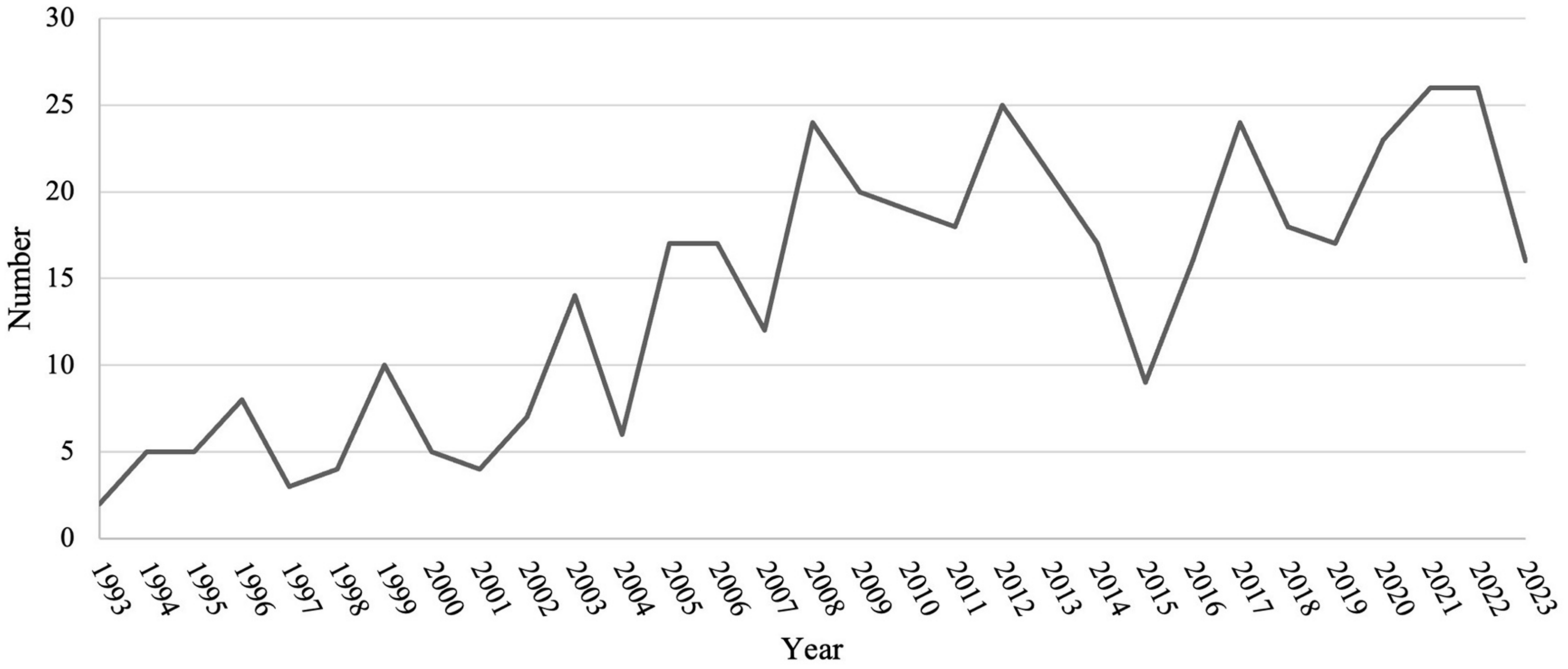
The number of publications from 1993 to 2023.

Figure 1 illustrates the process of searching and filtering. Initially, a database search was conducted in the Web of Science Core Collection using keywords mentioned in 2.1, from January 1, 1993, to October 31, 2023, resulting in 703 articles. These articles were then filtered by document type and language, focusing on articles, review articles, and book chapters written in English, narrowing down the pool to 594 articles. Further screening based on titles and abstracts refined to 438 records, which enters the final analysis stage. Figure 2 shows the number of publications every year from 1993 to 2023. Starting from 1993, papers in this field have been continuously developing, reaching their peak in 2022 and 2023 (*n* = 26). There was a noticeable decline in 2015 (*n* = 9), but in the following years, it remained in a fluctuating upward trend.

### 3.2 Analysis of authors’ country of origin

**Figure 3:**
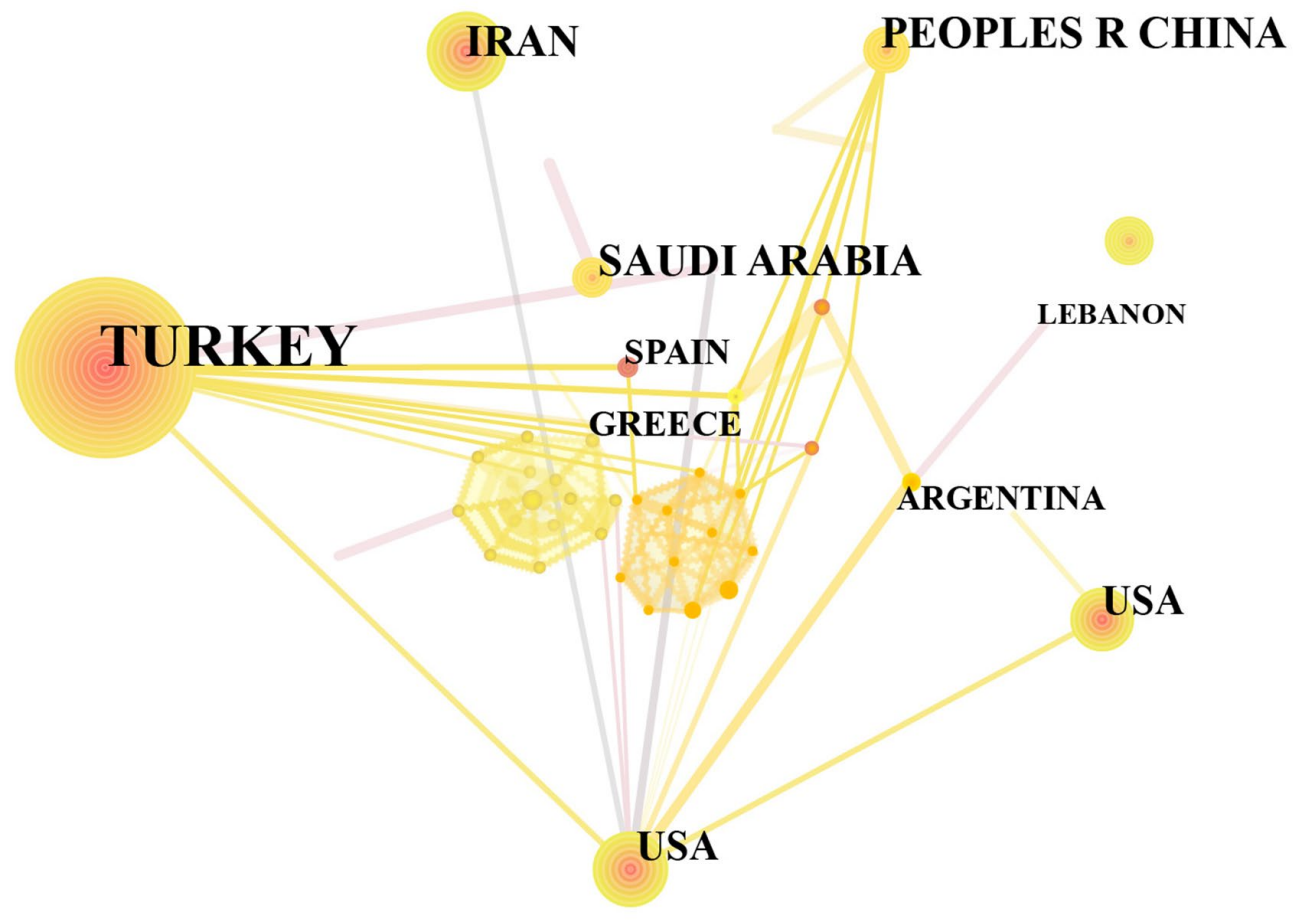
The map of country cooperation networks.

Examining the author’s nationality and national collaboration presents the geographical distribution of research and visualizes the extent of international cooperation, enhancing global collaborative networks and helping address geographic disparities in scientific output. In Citespace software, by selecting “Country” as the Node Type, there are 438 articles from 59 countries. Table 1 displays the top 10 countries in terms of the number of published papers. Figure 3 shows the national collaboration graph. Each node represents a country, with larger nodes indicating a higher number of published articles from that country. The lines between nodes signify collaboration among authors from different countries. As the top-ranking country, Turkey’s contribution exceeds 30% (*n* = 154), followed by Iran (*n* = 37) and India (n = 33). Turkey leads in research on Brucellosis and Neurological diseases, but its collaboration with other countries is relatively limited. Despite a relatively low publication volume, the United States exhibits the highest level of collaboration with other countries.

**Table 1:** The ranking of the top 10 contributed countries.

Rank	Country	Number	Percent
1	Turkey	154	34.5%
2	Iran	37	8.3%
3	India	33	7.4%
4	China	31	7.0%
4	United States	31	7.0%
6	Saudi Arabia	29	6.5%
7	Spain	13	2.9%
8	Greece	11	2.5%
9	Tunisia	11	2.5%
10	Argentina	11	2.5%

### 3.3 Analysis of universities and institutions

The analysis of universities and institutions serves as an indicator of research productivity. It identifies centers of excellence and guides potential collaborations and resource allocation to enhance research, diagnosis, and treatment strategies in this field. A total of 423 institutions have published research about neurobrucellosis. Table 2 shows the top 10 institutions in terms of the number of published papers. Gaziantep University, having published the highest number of articles in this field, has only contributed 12 papers. This suggests that few institutions are conducting in-depth research in this field. Apart from the seventh institution is from Argentina, the remaining nine institutions are all from Turkey.

**Table 2:** The ranking of the top 10 contributed universities and institutions.

Rank	Institutions	Number	Percentage
1	Gaziantep University	16	3.7%
2	Ankara Numune Training Research Hospital	14	3.2%
3	Gulhane Military Medical Academy	14	3.2%
4	Yuzuncu Yil University	14	3.2%
5	Baskent University	11	2.5%
6	Istanbul Haydarpasa Numune Training Research Hospital	11	2.5%
7	University Of Buenos Aires	10	2.3%
8	Erciyes University	9	2.1%
9	Istanbul Haydarpasa Sultan Abdulhamid Training Research Hospital	9	2.1%
10	Istanbul University	9	2.1%

### 3.4 Analysis of author cooperation

**Figure 4:**
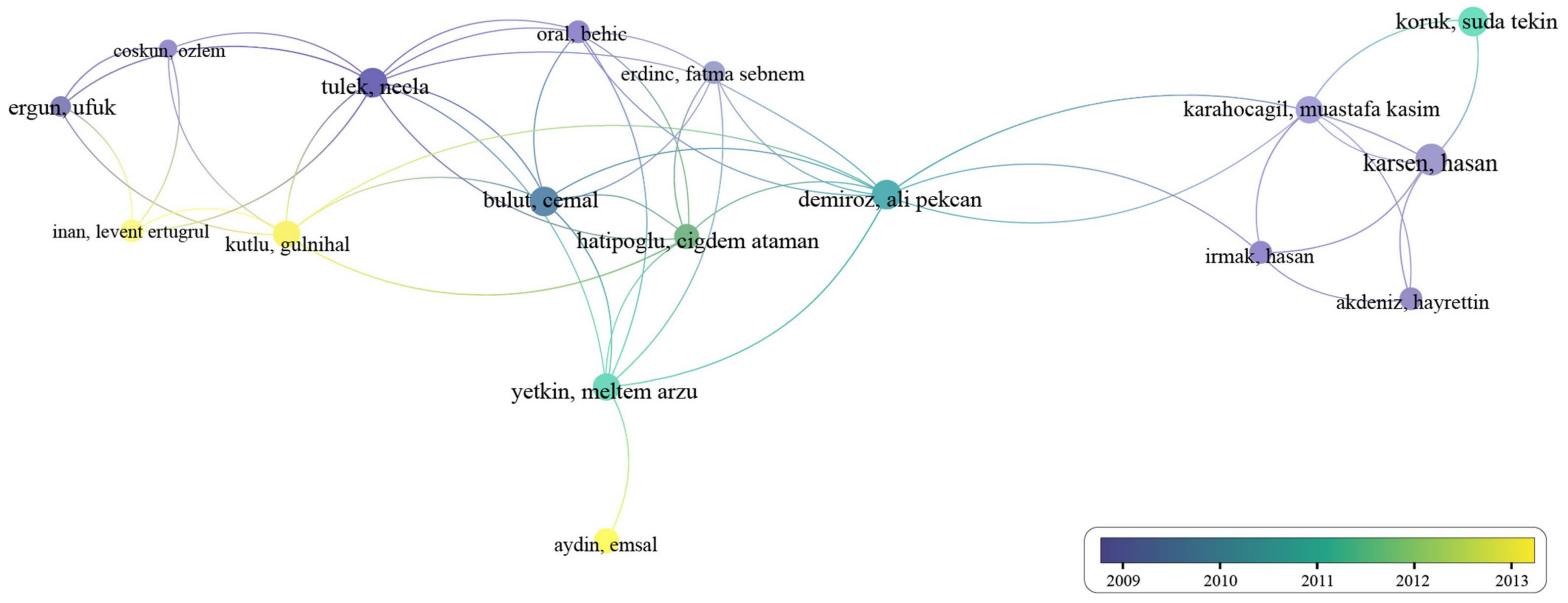
The map of co-authorship.

Analyzing author cooperation allows us to observe trends in collaboration frequency over different time periods, providing insights into how research communities evolve and adapt in response to emerging challenges and shifts in scientific focus. In VOSviewer software, choose “co-authorship” as the analysis type, “author” as the unit of analysis, and set the minimum number of documents for an author to be 2. There are a total of 153 authors that meet the criteria. Giambartolomei GH, Gul HC, and Namiduru M have the highest number of publications (*n* = 9), followed by Erdem H (*n* = 8), Delpino MV, Demiroglu YZ, and Karsen H (*n* = 7). Figure 4 shows the authors’ cooperation map. Each node represents an author. And the line that links the two nodes presents the collaboration between the two authors. Authors who have published two or more articles collaborated predominantly between 2009 and 2013, with the majority of collaborations occurring around 2010. Over the past decade, there has been almost no collaboration among authors finishing two or more co-authored articles.

### 3.5 Analysis of publications and journals

The analysis of publications and journals aims to identify the most influential platforms for neurobrucellosis research, helping researchers target journals that maximize the visibility and impact of their work. From 1993, January to 2023, October 31, there were 245 journals publishing articles related to the neurobrucellosis. Table 3 presents the top 10 journals with the most published articles on neurobrucellosis. Except for the fourth and ninth journals, which are relatively broad medical journals, the others are specialized in neurology or infectious diseases. Neurology published the highest number of articles (*n* = 12), followed by the European Journal of Neurology (*n* = 9), Clinical Infectious Diseases, Cureus Journal of Medical Science, and International Journal of Infectious Diseases (*n* = 8).

**Table 3:** The rank of top publication journals.

Rank	Journal	Number	Percentage
1	Neurology	12	2.74%
2	European Journal of Neurology	9	2.06%
3	Clinical Infectious Diseases	8	1.83%
4	Cureus Journal of Medical Science	8	1.83%
5	International Journal of Infectious Diseases	8	1.83%
6	Clinical Neurology and Neurosurgery	6	1.37%
7	Journal of Pediatric Infection	6	1.37%
8	Journal of the Neurological Sciences	6	1.37%
9	Archives of Iranian Medicine	5	1.14%
10	Case Reports in Infectious Diseases	5	1.14%

### 3.6 Analysis of co-citation

Co-citation analysis assesses the collective influence of articles by examining their relationships based on co-citation frequency, revealing underlying connections and emerging trends (24). It is significant for identifying key studies and related findings, as well as guiding future research directions. In VOSviewer software, choose “co-citation” as the analysis type, “cited reference” as the unit of analysis, and set the minimum number of citations of a cited reference to be 10. Among the 4,462 cited references, 121 meet the criteria. The c-cited articles are classified into three main clusters, which provide an overview of the previous research directions. Table 4 illustrates representative articles from the three primary clusters.

**Table 4:** Main co-cited literature information.

Main clusters	Authors	Title	Citations	Journal	DOI
#1 Therapy methods	McLean et al. (32)	Neurobrucellosis: Clinical and Therapeutic Feature	128	Clinical Infectious Diseases	10.1093/clind/15.4.582
	Shakir et al. (13)	Clinical categories of neurobrucellosis. A report on 19 cases	130	Brain: a Journal of Neurology	10.1093/brain/110.1.213
#2 Diagnosis of neurobrucellosis	Gul et al. (1)	Overview of neurobrucellosis: a pooled analysis of 187 cases	75	International Journal of Infectious Diseases	10.1016/j.ijid.2009.02.015
	Guven et al. (12)	Neurobrucellosis: clinical and diagnostic features	73	Clinical Infectious Diseases	10.1093/cid/cit072
#3 Overview of Brucellosis and its complications	Bodur et al. (74)	Neurobrucellosis in an Endemic Area of Brucellosis	45	Scandinavian Journal of Infectious Diseases	10.1080/0036554021000027000
	Buzgan et al. (75)	Clinical manifestations and complications in 1028 cases of brucellosis: a retrospective evaluation and review of the literature	44	International Journal of Infectious Diseases	10.1016/j.ijid.2009.06.031

**Figure 5:**
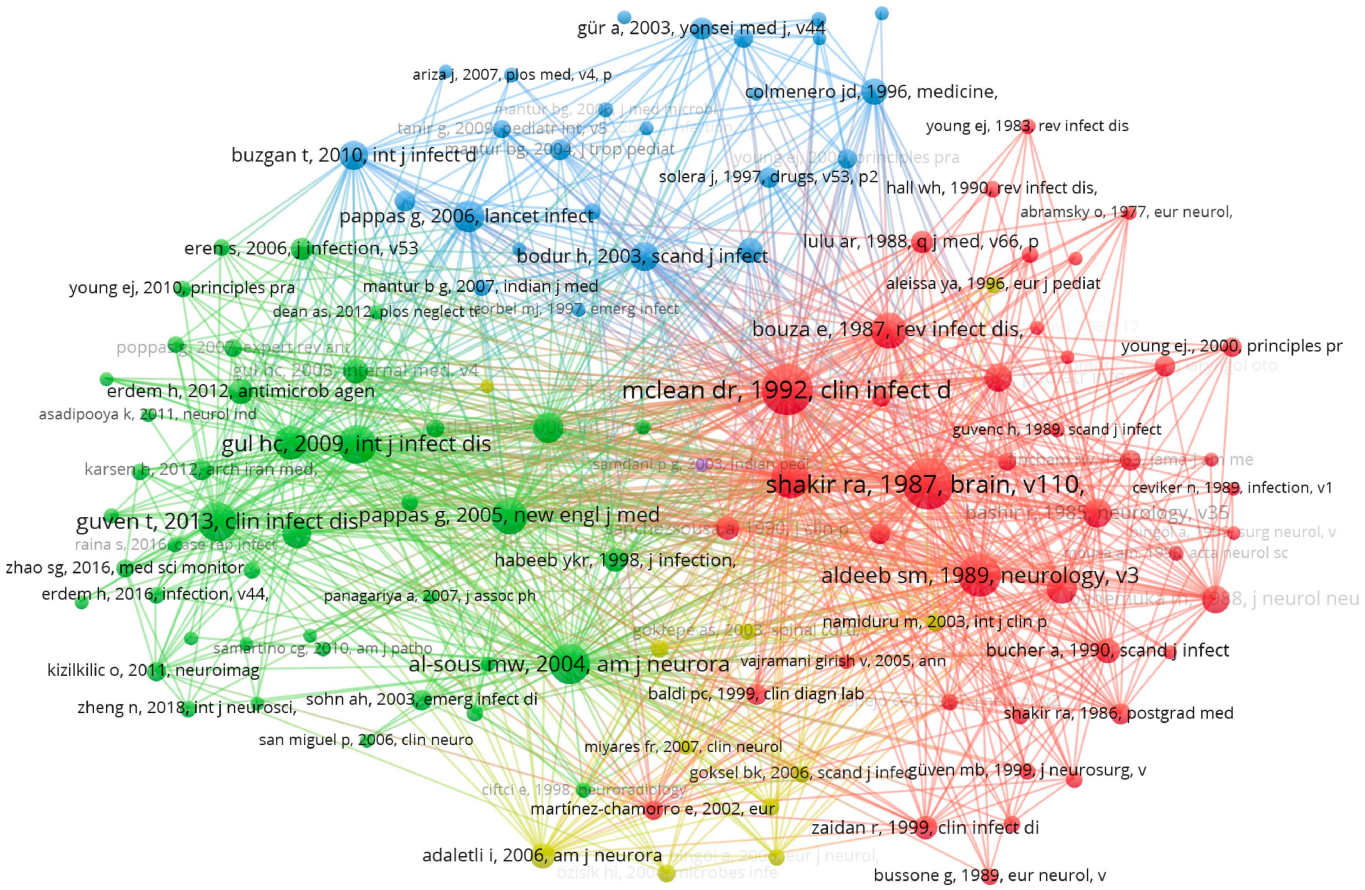
The map of co-citation.

Figure 5 shows the map of co-citation. The red nodes represent the first cluster, in which “Neurobrucellosis: Clinical and Therapeutic Feature,” and “Clinical categories of neurobrucellosis. A report on 19 cases” are the most cited articles, all of which describe the therapy of neurobrucellosis. The green dots represent the second cluster, where “Neurobrucellosis: clinical and diagnostic features,” and “Overview of neurobrucellosis: a pooled analysis of 187 cases” are representative articles about the diagnosis of neurobrucellosis. Some of the articles in this cluster overlap with the first cluster. The blue nodes represent the third cluster. “Neurobrucellosis in an Endemic Area of Brucellosis” and “Clinical Manifestations and Complications in 1028 Cases of Brucellosis: A Retrospective Evaluation and Review of the Literature” are two contributed articles providing an overview of Brucellosis and its complications, clinical manifestations, and therapy methods.

“Overview of neurobrucellosis: a pooled analysis of 187 cases” and “Neurobrucellosis: clinical and diagnostic features” are the two most cited articles on the diagnosis of neurobrucellosis. Gul et al. (1) evaluated 187 cases of neurobrucellosis and found that the main symptoms in patients were headache, fever, sweating, and weight loss, and the major complications included cranial nerve involvement, polyneuropathy, depression, paralysis, stroke, and abscess formation. The low incidence rate and diverse presentations pose a great challenge to the diagnosis of neurobrucellosis (1, 3). Neurological complications of brucellosis could manifest at any stage, but the diagnosis is typically made 2–12 months after symptom onset (12). The study made by Guven et al. (12) indicates that Brucella tube agglutination (TA) with the Coombs test, setting the cutoff greater than or equal to 1:8, is highly sensitive and specific, and it enables the rapid detection of patients with Brucella in the cerebrospinal fluid.

“Neurobrucellosis: Clinical and Therapeutic Feature” and “Clinical categories of neurobrucellosis. A report on 19 cases” are two main cited articles related to the therapy of neurobrucellosis. Shakir et al. (13) described 19 cases of neurobrucellosis and classified them into three groups based on clinical presentations: acute meningoencephalitis presentation, chronic central presentation, and chronic peripheral presentation. The pathology and treatment responses vary among different categories. For example, the acute meningoencephalitis form is marked by severe, acute inflammation of the meninges and brain tissue, often accompanied by cerebral edema and granulomatous inflammation, leading to rapid onset symptoms such as headaches, fever and seizures (25–27). In contrast, the chronic central presentation involves prolonged granulomatous inflammation within the central nervous system, resulting in a more insidious onset with cognitive decline and neuropsychiatric disturbances, and a higher risk of relapse (12, 27–29). The chronic peripheral form is characterized by less intense inflammation but is often more resistant to standard treatments, presenting with persistent neuropathic symptoms like numbness, tingling, and muscle weakness (30, 31). Proper awareness and serological testing are essential to differentiate neurobrucellosis from other chronic CNS infections, particularly tuberculosis, and neurosyphilis (13). The standard treatment for brucellosis includes streptomycin and tetracycline (5, 7, 32). However due to the limited penetration of these drugs through the blood–brain barrier, additional antimicrobial agents like rifampin and TMP-SMZ, capable of reaching the cerebrospinal fluid (CSF), are necessary for treating central nervous system involvement (32). McLean et al. (32) recommend a combination of three or four antimicrobial agents in curing neurobrucellosis and suggested treatment duration ranges from 2 to 4 months, contingent upon the patient’s clinical and CSF responses.

### 3.7 Analysis of keyword mapping

**Figure 6:**
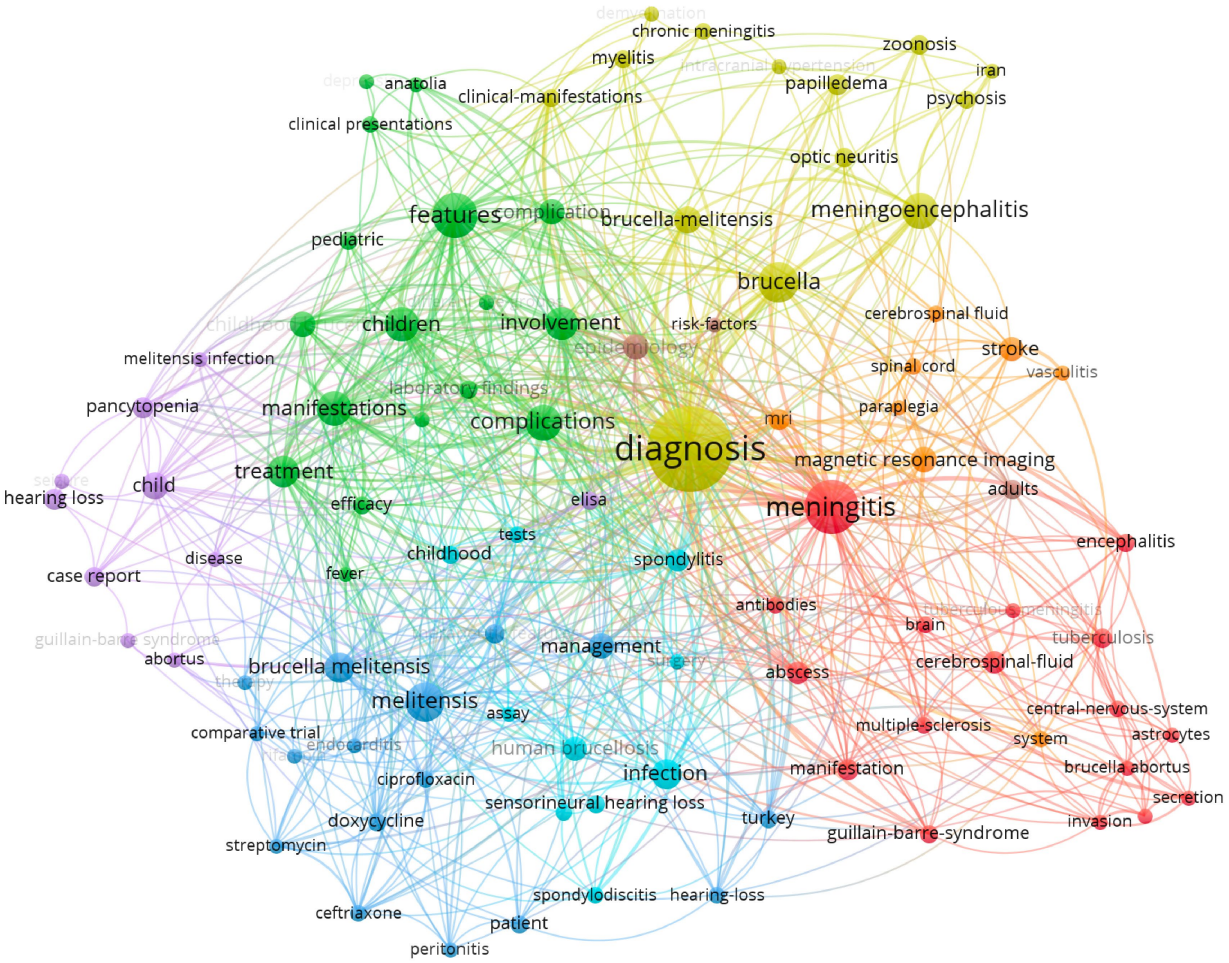
The map of keywords co-occurrence.

Keyword mapping visualizes the relationships between individual keywords, highlighting connections across the research field. In VOSviewer software, by selecting “Co-occurrence” as the type of analysis and “All Keywords” as the unit of analysis and setting the minimum number of occurrences of a keyword as 4, there are 98 keywords. Figure 6 presents the network mapping based on the frequency of keywords. Table 5 shows the top 15 keywords in terms of frequency except the keywords related to the topic like “Brucellosis,” and “neurobrucellosis.”

**Table 5:** The ranking of the top 15 keywords.

Rank	Keyword	Occurrences	Total link strength
1	Diagnosis	121	497
2	Meningitis	50	215
3	Features	34	165
4	Complications	23	115
5	Meningoencephalitis	22	81
6	Manifestations	21	106
7	Children	20	91
8	Involvement	19	115
9	Treatment	17	91
10	Infection	16	55
11	Childhood brucellosis	12	53
12	Epidemiology	11	59
13	Magnetic resonance imaging	11	42
14	Management	11	42
15	Stroke	10	33

Based on the popular keywords the research focus in this field primarily revolves around diagnosis features and manifestation which reflects that research is more towards reporting the basic information of cases. Meningitis is the most commonly mentioned neurological disease associated with brucellosis

### 3.8 Analysis of keywords cluster

**Figure 7:**
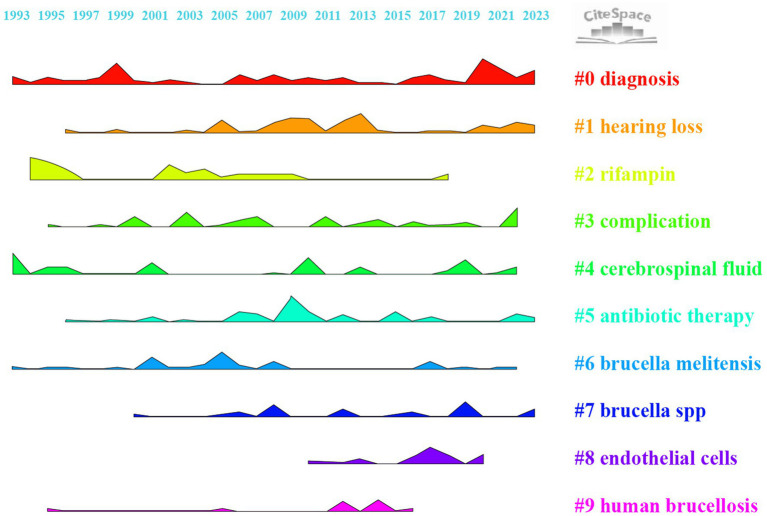
Ridgeline plot for keyword cluster analysis.

Keyword clustering organizes the related keywords into thematic groups, offering a more detailed analysis of specific research topics or subfields. While keywords mapping shows how concepts are linked, clustering defines and categorizes these connections into coherent research themes. In CiteSpace software, keywords cluster analysis applies the Log-likelihood ratio (LLR) algorithm. Table 6 shows the basic information of the clusters. The network parameters, Modularity Q = 0.51 > 0.3, Weighted Mean Silhouette S = 0.81 > 0.7, and Harmonic Mean (Q, S value) = 0.62 > 0.5 indicate that the clustering results have a high degree of credibility. Figure 7 shows how nine keywords have evolved from 1993 to 2023. #0 Diagnosis and #4 Cerebrospinal Fluid are popular topics, being the earliest to appear and continuing until recent times. #1 Hearing Loss, #5 Antibiotic Therapy, and #7 Brucella spp. have also continued to appear until recent years. #4 Cerebrospinal Fluid and #5 Antibiotic Therapy clusters have not shown continuous spikes, which may indicate potential future research directions.

**Table 6:** Keywords clusters information.

Cluster	Size	Top five keywords
#0 Diagnosis	88	Diagnosis (16, 1.0E-4); meningitis (12.2, 0.001); children (11.79, 0.001); nervous system brucellosis (9.75, 0.005); magnetic resonance imaging (9.75, 0.005)
#1 Hearing Loss	54	Hearing loss (18.87, 1.0E-4); case report (15.7, 1.0E-4); abducens nerve palsy (6.25, 0.05); diagnosis and treatment (6.25, 0.05); neuropathy (6.25, 0.05)
#2 Rifampin	44	Rifampin (15.63, 1.0E-4); comparative trial (15.63, 1.0E-4); regimen (15.63, 1.0E-4); tetracycline streptomycin (10.39, 0.005); plus streptomycin (10.39, 0.005)
#3 Complication	43	Complication (11.4, 0.001); laboratory findings (8.43, 0.005); clinical features (8.43, 0.005); epidemiology (6.46, 0.05); treatment (5, 0.05)
#4 Cerebrospinal Fluid	43	cerebrospinal fluid (16.36, 1.0E-4); central nervous system (11.51, 0.001); *mycobacterium tuberculosis* (10.54, 0.005); whipples disease (10.54, 0.005); polymerase chain reaction (10.54, 0.005)
#5 Antibiotic Therapy	41	Antibiotic therapy (10.31, 0.005); mr imaging (5.14, 0.05); spinal epidural abscess (5.14, 0.05); knowledge (5.14, 0.05); rat (5.14, 0.05)
#6 *Brucella Melitensis*	31	*Brucella melitensis* (14.63, 0.001); guillain-barre syndrome (7.34, 0.01); brucella endocarditis (5.49, 0.05); nf-kappa b p65 (5.49, 0.05); autophagy (5.49, 0.05)
#7 Brucella spp.	24	Brucella spp. (13.98, 0.001); immigrant (6.94, 0.01); cytomegalovirus (6.94, 0.01); protean manifestations (6.94, 0.01); serological surveillance (6.94, 0.01)
#8 Endothelial Cells	23	Endothelial cells (15.51, 1.0E-4); *brucella abortus* (10.05, 0.005); dendritic cells (7.69, 0.01); nlrp3 (7.69, 0.01); blood–brain barrier (7.69, 0.01)
#9 Human Brucellosis	12	Human brucellosis (11.24, 0.001); scrofula (7.45, 0.01); pcr (7.45, 0.01); samuel johnson (7.45, 0.01); cervical lymphadenitis (7.45, 0.01)

### 3.9 Analysis of burst detection

**Figure 8:**
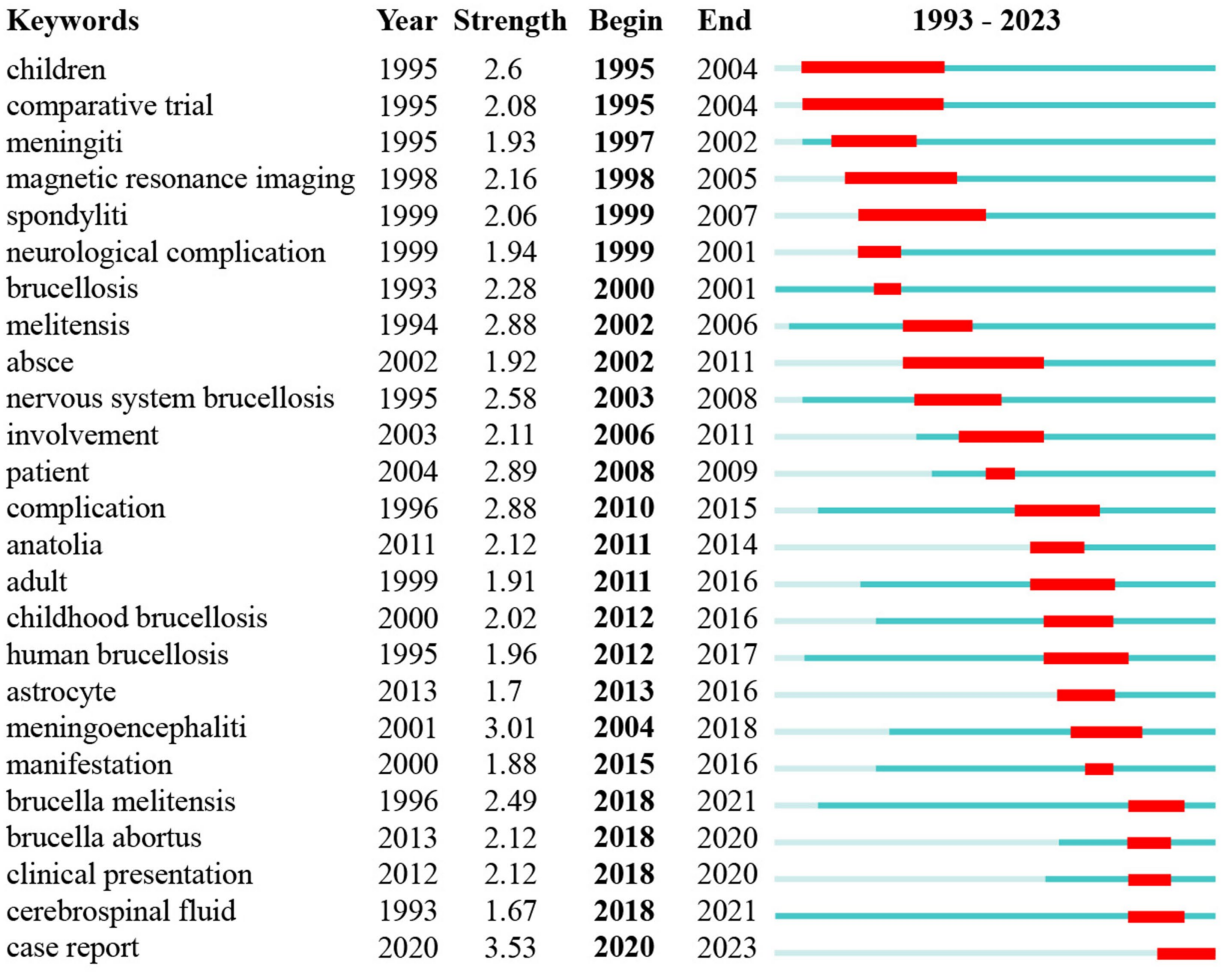
The ranking of the top 25 keywords with the strongest citation bursts.

Keyword burst analysis is conducted to identify periods when specific keywords experience a sudden increase in frequency, indicating emerging trends or shifts in research focus (33). This method helps to pinpoint when certain topics become highly relevant, often correlating with discoveries, technological advancements, or changes in societal concerns. By identifying these “bursts,” researchers can gain insights into the dynamics of a field, track the evolution of interest in specific topics, and predict future research directions. Building on the information from section 3.7, a keyword burst analysis was conducted. Figure 8 displays the top 25 burst keywords for brucellosis and neurological diseases from 1993 to 2023. The keyword with the highest burst strength is “case report” (Str = 3.53), followed by “meningoencephalitis” (Str = 3.01). The keyword with the longest burst duration is “children” (Year = 9), followed by “comparative trial” (Year = 8). “Cerebrospinal fluid” emerged as early as 1993 but experienced a surge starting in 2018. This suggests that prior research on this topic was relatively shallow and not extensive. It will highly likely become a popular keyword for future research.

## 4 Discussion

Neurobrucellosis is a neurological complication of brucellosis and demonstrates diverse neurological manifestations. The most common presentation is acute meningitis, with symptoms such as headache, fever, and neck stiffness (6, 25, 34). Meningoencephalitis is also frequently observed, involving prolonged inflammation of the brain and its membranes (25, 35). Chronic radiculoneuropathy, another syndrome of neurobrucellosis, involves inflammation of the nerve roots and peripheral nerves, leading to pain and sensory disturbances (36). Less common but notable presentations include pseudotumor cerebri, where increased intracranial pressure mimics symptoms of a brain tumor, and myelopathy, which affects the spinal cord leading to motor and sensory impairments (37, 38). These syndromes emphasize the complexity of neurobrucellosis, requiring prompt and effective management strategies.

This research focused on the statistical analysis of the 438 articles from Web of Science Core Collaboration. The existing articles in this field predominantly consist of case reports and most of them have relatively small sample sizes (14–16, 39). Researchers found a general upward trend in the articles about neurobrucellosis during the past 30 years. Turkey leads in the number of publications on neurobrucellosis, contributing over 30% of the total articles, with nine out of the top 10 institutions in this field also based in Turkey. However, despite the high volume of research, these Turkish institutions have not conducted in-depth studies in this field. In contrast, the University of Buenos Aires in Argentina has been conducting significant immunopathological research, accounting for 10 of the 11 articles from Argentina (18, 40–43). They shed light on neurobrucellosis and contributed valuable insights to the field. Author collaborations, with two or more articles, were mostly concentrated around 2010, with few collaborations in the past decade. The journal “Neurology” published the highest number of articles related to brucellosis and neurological disease. From the analysis of co-citations, articles related to neurobrucellosis are generally categorized into three main groups: therapy methods, diagnosis of neurobrucellosis, and overview of Brucellosis and its complications, which indicated the past research directions. “Diagnosis,” “meningitis,” and “features” are the most frequently occurring keywords, indicating that articles in this field predominantly focus on case reports, and the most frequently reported neurological complication is meningitis. Researchers conducted a cluster analysis of the keywords and found that “Cerebrospinal Fluid and Antibiotic” and “Antibiotic Therapy” have research value, as they have been consistently appearing in recent years, whereas there was no sustained outbreak of these topics in the earlier years. The burst analysis of the keywords also indicated that “cerebrospinal fluid” may become a prominent keyword in future research. The term emerged as early as 1993 but only began to gain prominence in 2018, indicating its research significance.

Cerebrospinal fluid (CSF) is emerging as a focal point for future research, with a primary focus on the detection of neurobrucellosis. Neurobrucellosis poses diagnostic challenges due to non-specific clinical manifestations and inefficient routine culture tests (44). Traditional approaches to detecting neurobrucellosis, such as microbial culture and serological tests, have limitations in application due to low sensitivity or specificity, along with the requirement for a long sample-to-answer turnaround time (45–47). Serological tests include RBPT (Rose Bengal plate agglutination tests), STAT (Standard tube agglutination test), and ELISA (Enzyme-linked immunosorbent assay) to identify antibodies in serum and CSF (1, 2). PCR (Polymerase chain reaction) for specific DNA sequences and oligoclonal immunoglobulin G (IgG) tests in cerebrospinal fluid are also traditional methods to confirm neurobrucellosis (48–50). Using a combination of diagnostic and clinical methods is recommended for accurate brucellosis detection. Recent research found that Next-generation sequencing (NGS) is a rising method for identifying pathogenic microorganisms based on sequencing, and its utilization is growing in the diagnosis of infectious diseases affecting the central nervous system (CNS) (46, 51). Next-generation sequencing (NGS) is also recommended to be used in diagnosing neurobrucellosis due to its Enhanced Sensitivity and rapid Diagnosis (44, 45, 52). Other research proposed that Metabolites of cerebrospinal fluid (CSF) are biomarkers for neurobrucellosis, representing a potential diagnostic modality for neurobrucellosis (53, 54). CSF samples from neurobrucellosis patients and normal controls were analyzed using liquid chromatography-mass spectrometry (LC–MS) to detect metabolites. The results showed differences in metabolite profiles between neurobrucellosis patients and normal controls, and elevated inflammatory cytokines in CSF further support the utility of metabolomics for diagnosis and treatment insights (53). Although the effectiveness of Next-Generation Sequencing (NGS) and the identification of metabolites of cerebrospinal fluid using LC–MS for detecting neurobrucellosis has been proven, large-scale studies with expanded sample sizes are crucial for further validation (44). Further research into the diagnosis of neurobrucellosis is expected to lead to earlier and more precise identification of the condition, which enables timely and appropriate therapeutic interventions. It is also crucial to validate the efficacy of these diagnostic methods and establish standardized protocols for their use in clinical settings.

Magnetic Resonance Imaging (MRI) can be also instrumental in diagnosing neurobrucellosis. A study classified the MRI features of neruobrucellosis into five types: In meningitis (Type I), MRI often displays equal T1 and high T2 signal intensities, accompanied by meningeal-like enhancement, primarily affecting the frontal and temporal lobes (55). Meningoencephalitis (Type II) is characterized by abnormal signal intensities in the meninges and submeningeal brain regions, presenting with similar T1 and elevated T2 signals. The presence of subtle meningeal enhancements without significant submeningeal tissue alteration further aligns with findings from specialized case studies (55). Inflammatory demyelinating (Type III) features diffuse myelitis, increased intensity in the cerebral white matter, and localized white matter changes (55, 56). These alterations are typically found around critical structures such as the lateral ventricles and spinal cord (55–57). Ata et al. (58) also reported that MRI characteristics of demyelination include meningeal enhancement, particularly around the brainstem and along cranial nerves and the lower cranial nerves. The Abscess (Type IV) presents enhancement both in the brain and spinal cord similar to the typical abscess. Pseudotumor (Type V) mimics neoplastic processes with well-defined, enhancing lesions that exhibit low T1 and high T2 signals, demanding meticulous differential diagnosis (55).

Antibiotic therapy is commonly used to treat neurobrucellosis. Shaw et al. (59) advocate for multiagent antibiotic protocols, specifically incorporating ceftriaxone, rifampin, and doxycycline, as the preferred therapeutic approach. They also recommended a treatment duration of 4–6 weeks for ceftriaxone and a minimum of 12 weeks for rifampin and doxycycline, underscoring the pivotal role of strict adherence in mitigating the risk of relapse (59) Many studies have also confirmed the efficacy of antibiotic therapy in various neurological complications of brucellosis. For example, protracted polymicrobial antibiotic therapy and heparin are also proven to be effective in achieving a favorable outcome in patients with neurobrucellosis accompanied by cerebral venous sinus thrombosis (CVST) (60). Antibiotic regimen involving rifampicin, doxycycline, and trimethoprim/sulfamethoxazole is effective for neurobrucellosis complicated with sensorineural hearing loss (SNHL) (15). However, antibiotic therapy may not be permanently effective. Mirza et al. (15) mentioned a previous case report where SNHL persisted even after 6 weeks of antibiotic treatment, which suggested future research about the relationship between neurobrucellosis and SNHL. A documented case of neurobrucellosis with clinical manifestations resembling amyotrophic lateral sclerosis (ALC) reported that the disease-specific antibiotic therapy failed to impede the progression of motor neuron disease, suspecting a causal association between systemic neurobrucellosis and ALS (49).

The primary treatment for neurobrucellosis is antibiotics, but corticosteroids are also used in some situations to mitigate the damaging effects of bacterial toxins, potentially reducing the likelihood of enduring complications (6). Evidence from case reports suggests that corticosteroid therapy can effectively alleviate neurological symptoms such as headaches and vision problems and moderate the inflammatory response in neurobrucellosis patients (61). Despite these benefits, the efficacy of corticosteroids has not been conclusively established through large-scale studies or randomized controlled trials. Recent research indicates a significant concern: corticosteroid therapy was associated with a higher risk of sequelae or relapse (62). It contrasts with outcomes in other conditions like Pneumococcal meningitis, where steroid use is associated with a reduced risk of neurological sequelae, including hearing loss (63). Consequently, while corticosteroids can provide symptomatic relief in neurobrucellosis, the potential risks must be carefully weighed against the benefits.

The relationship between neurobrucellosis and neurological conditions arises from the frequent occurrence of neurological diseases among patients with brucellosis (41). The underlying mechanisms of neurobrucellosis involve direct invasion by *Brucella abortus* into the Central Nervous System (CNS), primarily facilitated through two routes: the “Trojan horse” mechanism and transcellular traversal of the blood–brain barrier (BBB). *B. abortus* utilizes infected monocytes to cross the BBB, effectively employing these cells as a means to enter the CNS (64, 65). Once inside, the bacteria can infect brain microvascular endothelial cells (HBMEC), as well as resident CNS cells like astrocytes and microglia (65). The presence of the bacteria triggers an inflammatory response, characterized by the production of cytokines and chemokines, which leads to astrogliosis and potentially to the apoptosis of astrocytes, contributing to the pathology observed in neurobrucellosis (42). The hypothesis is that the bacterial antigens may mimic neuronal antigens (molecular mimicry), leading to an autoimmune response that targets the myelin sheath of nerve cells (6). This could explain the demyelinating-like lesions observed in some patients with neurobrucellosis (66). Both neurobrucellosis and demyelinating disorders such as multiple sclerosis (MS) can present with neurological symptoms like headaches, visual disturbances, ataxia, and sensory or motor deficits (6, 12, 67). MRI findings in neurobrucellosis often include meningeal enhancement, white matter lesions, and other signs of inflammation which are also commonly seen in demyelinating disorders (66, 68). These similarities necessitate a careful differential diagnosis to distinguish between infectious causes and primary demyelinating processes. Neurobrucellosis is also reported to potentially link to psychiatric disorders, a connection supported by its inflammatory and systemic impacts on the central nervous system, which may lead to neuropsychiatric symptoms (69). Studies have shown that patients with neurobrucellosis experience significant emotional disturbances, and exhibit severely impaired cognitive functions, such as mental control, logical memory, and visual reproduction, and score higher on measures of depressive symptoms compared to control subjects (1, 70, 71). Further research needs to clarify these associations and understand the underlying pathophysiological processes, although some reports have recently begun to investigate the pathophysiological processes that associate psychiatric disorders with inflammatory responses elicited by the bacterium (72, 73). Understanding the complex interactions between neurobrucellosis and neurological diseases is crucial for improving diagnostic accuracy and patient outcomes.

This study has certain limitations. It exclusively used the Web of Science Core Collection Database for data analysis and focused on English-language articles during the screening process. This approach might lead to the omission of relevant studies. To counteract these limitations and strengthen the validity of our conclusions, we conducted a thorough review of additional key articles from prominent databases such as PubMed and Scopus in the discussion section, which broadened the scope and enhanced the comprehensiveness of the analysis.

This article employed both CiteSpace and VOSviewer software to make a bibliometric analysis of articles related to neurobrucellosis. By identifying the most active regions, leading institutions, and prevalent themes in current research, this analysis outlines the intellectual landscape and gaps, and guides researchers to develop targeted therapies and interventions that address the most pressing needs in the management of Neurobrucellosis, enhancing clinical outcomes and patients’ quality of life.

## Data Availability

The original contributions presented in the study are included in the article/supplementary material, further inquiries can be directed to the corresponding author.
